# Enhancer analysis of the *Drosophila* zinc finger transcription factor Earmuff by gene targeting

**DOI:** 10.1186/s41065-021-00209-6

**Published:** 2021-11-04

**Authors:** Kirsten Hildebrandt, Sabrina Kübel, Marie Minet, Nora Fürst, Christine Klöppel, Eva Steinmetz, Uwe Walldorf

**Affiliations:** 1 Developmental Biology, Saarland University, Building 61, 66421 Homburg/Saar, Germany; 2 Present address: Clinical and Molecular Virology, Friedrich-Alexander University, 91054 Erlangen, Germany; 3 Present address: Human Genetics, Saarland University, Building 60, 66421 Homburg/Saar, Germany; 4 Present address: Genetics/Epigenetics, Saarland University, Building A2.4, 66123 Saarbrücken, Germany; 5 Present address: Zoology and Physiology, Saarland University, Building B2.1, 66123 Saarbrücken, Germany

**Keywords:** Earmuff (Erm), Transcription factor, Enhancer, Gene targeting

## Abstract

**Background:**

Many transcription factors are involved in the formation of the brain during the development of *Drosophila melanogaster*. The transcription factor Earmuff (Erm), a member of the forebrain embryonic zinc finger family (Fezf), is one of these important factors for brain development. One major function of Earmuff is the regulation of proliferation within type II neuroblast lineages in the brain; here, Earmuff is expressed in intermediate neural progenitor cells (INPs) and balances neuronal differentiation versus stem cell maintenance. Erm expression during development is regulated by several enhancers.

**Results:**

In this work we show a functional analysis of *erm* and some of its enhancers. We generated a new *erm* mutant allele by gene targeting and reintegrated Gal4 to make an *erm* enhancer trap strain that could also be used on an *erm* mutant background. The deletion of three of the previously analysed enhancers showing the most prominent expression patterns of *erm* by gene targeting resulted in specific temporal and spatial defects in defined brain structures. These defects were already known but here could be assigned to specific enhancer regions*.*

**Conclusion:**

This analysis is to our knowledge the first systematic analysis of several large enhancer deletions of a *Drosophila* gene by gene targeting and will enable deeper analysis of *erm* enhancer functions in the future.

**Supplementary Information:**

The online version contains supplementary material available at 10.1186/s41065-021-00209-6.

## Background

The *Drosophila earmuff* (*erm*) gene encodes a zinc finger transcription factor homologous to forebrain embryonic zinc finger proteins (Fezf) [1, 2] and therefore is a member of the Erm/FezF gene family [3]. Erm expression starts in early embryos with an anterior expression pattern resembling an earmuff [4]. In later embryonic stages expression is restricted to the brain, and brain specific expression is also visible during larval stages up to the adulthood. It was shown that Erm is one of several transcription factors that are important for the proliferation of type I and type II neuroblasts in the brain leading to an expansion of the brain region compared with the ventral nerve cord [5]. In *Drosophila* the brain is built by approximately 100 bilaterally arranged lineages [6–9]. Each lineage derives from a neuroblast, a neural stem cell dividing asymmetrically and thereby generating another neuroblast, and a neuronal precursor cell, the ganglion mother cell (GMC), through self-renewal. The GMC then divides symmetrically and produces two neurons. Through this mode of division, the neuroblast produces embryonic lineages of primary neurons [10]. This type of division is typical for type I neuroblasts that make up most of the cell lineages in the embryonic brain. In contrast to type I neuroblasts, type II neuroblasts generate intermediate neural progenitor cells (INPs) that divide 8–10 times to generate GMCs, which in turn divide into two neurons [11–13] thereby generating larger lineages. At the end of embryogenesis, most neuroblasts undergo a period of quiescence and resume their division during the larval stage [14]. In this postembryonic phase secondary neurons develop that make 90% of adult neurons [15]. In the larval brain all neuroblasts generate larger lineages compared to the embryonic brain, and type I lineages produce a progeny of approximately 100 neurons, while the eight type II lineages produce even up to 400 neurons [16]. Six of the eight type II lineages are located in the dorsomedial region, and the other two are located in the dorsolateral brain region. Earmuff is expressed in INPs of type II lineages and restricts the potential of these cells to proliferate more than normal [3]. If the *erm* function is lost in mutants such as *erm*^1^ and *erm*^2^, INPs dedifferentiate back into neuroblasts and a dramatic increase in neuroblasts and lineages is generated leading to enlarged brain hemispheres [3]. Therefore, Erm functions as a transcriptional repressor balancing neuronal differentiation versus stem cell maintenance. To restrict the progenitor cell potential Erm attenuates the competence of these cells to respond to self-renewal factors such as Deadpan (Dpn), Klumpfuss (Klu), Enhancer of split mγ [E (spl)mγ] and Notch (N) [17]. In this process, Erm functions after Brain tumour (Brat) and Numb and is expressed in immature INPs but not in mature INPs [17]. The BAP chromatin-remodelling complex, an association of the core Brahma complex with Osa [18, 19], most likely functions in parallel to Erm in this restriction process with immature INPs [17]. More recently, it was shown that the histone deacetylase Hdac1/Rpd3 [20] also functions in this way [21].

An interesting question is how the complex expression patterns of Erm are established and maintained over time. It is well accepted that the expression of genes in specific domains or tissues is regulated by sets of regulatory elements, among them enhancers, that can act over very large distances. The analysis of such elements in *Drosophila* is usually performed using reporter gene assays with lacZ or GFP as reporter genes ([22] for review). In the course of the *Drosophila* genome project more systematic attempts were made to identify enhancers of genes with known expression or functions in the adult brain [4]. To do so overlapping sequences of 3 kb upstream, downstream or in intronic regions of 925 genes were cloned in front of a Gal4 gene. More than 5000 transgenic fly strains were established, and the expression pattern of putative enhancers was analysed with the use of reporter genes in different developmental stages and tissues [23–25]. Another goal was to generate strains with small and well-defined expression domains that could be used to map specific brain areas. Integration of the constructs into the same chromosomal position allowed a direct comparison of the enhancer activities avoiding position effects. In the course of these analyses, *erm* was one of the first genes to be analysed in detail [4]. It was shown that five overlapping fragments from the 5′ region and two from the 3′ region of *erm* generate specific patterns in the embryo, larval brain and adult brain. The constructs R9D10 and R9D11 are of special interest, since they contain enhancer regions necessary for the expression in larval INPs [4, 21]. In particular, the overlapping region and directly neighbouring sequences in R9D11 were therefore analysed. It was shown that bHLH-O proteins such as Deadpan (Dpn) and E(spl) proteins which are expressed in neuroblasts, bind there and could suppress the expression of Erm [26]. The analysis of such strains to define enhancer regions of the *erm* gene would be a definitive step forward to understand the complex regulation of the gene, but a functional analysis of such regulatory elements might be a major goal for the future ([27] for review). This could be done by performing precise deletions of individual regions using gene targeting which was first established in mice ([28] for review) and later in *Drosophila* [29–31]. A more recently developed technique to mutate genes and to generate deletions of genomic regions is the CRISPR/Cas9 system [32, 33] which also functions in *Drosophila* [34–36].

In this paper we used the gene targeting technique in a first step to generate a new *erm* allele with a deletion of 1.5 kb, including the coding part of the second exon with the ATG and an alternatively used ATG in exon 3. By reintegration of Gal4 at that position, an *erm* enhancer trap strain was constructed and analysed. Through gene targeting we also made individual deletions of defined enhancer regions necessary for the expression of Erm in INPs of type II lineages, the optic lobes, the mushroom body and the intercerebral bridge and analysed their effects on the expression and functions of *erm*. Our findings reveal an important function of *erm* in various processes of *Drosophila* brain development.

## Results

### Generation of an *erm* mutant strain with reintegration of Gal4 in the *erm* locus

**Fig. 1:**
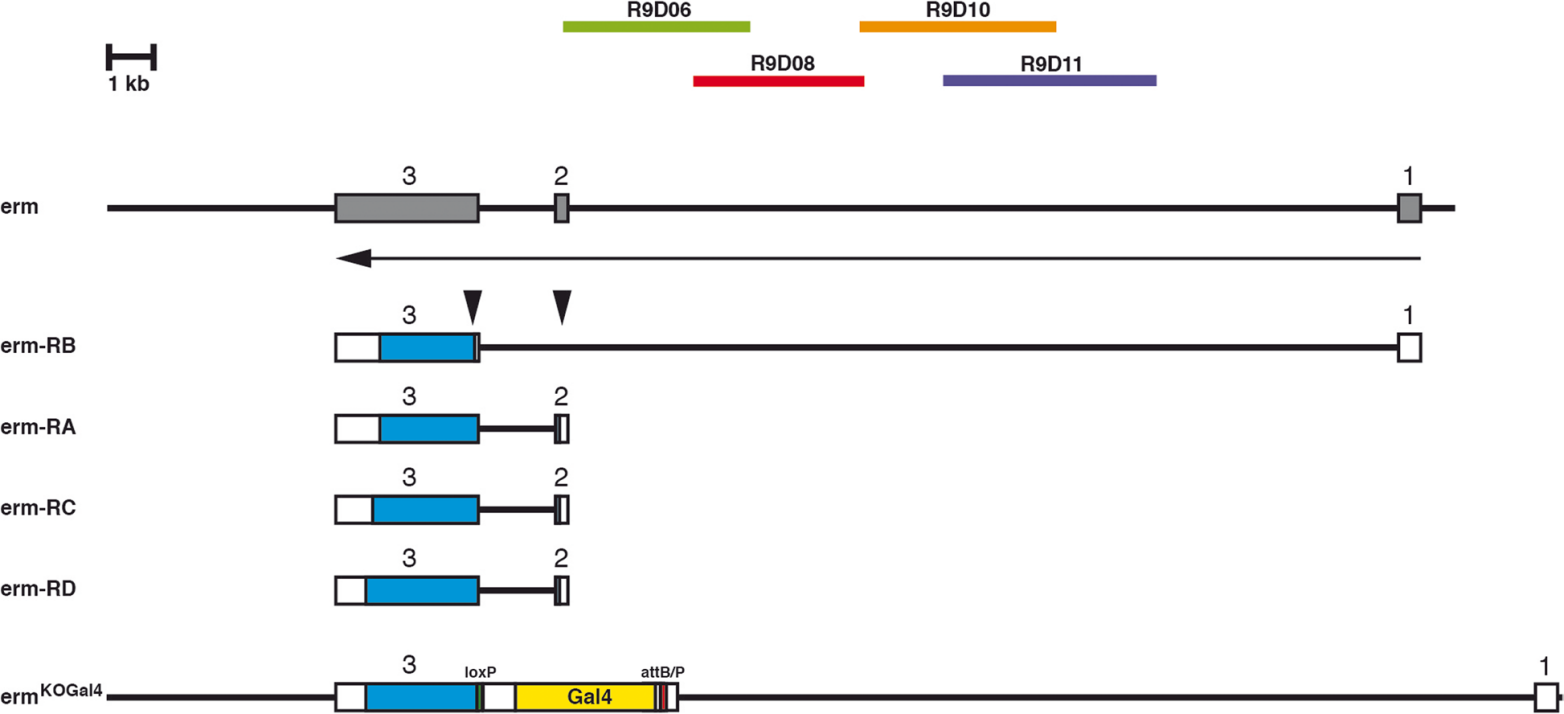
Generation of the erm^KOGal4^ strain. Genomic organization of the *erm* gene with exons indicated as grey boxes. The direction of transcription is shown by an arrow. The location of fragments used for the later analysis of enhancer activities is also indicated (Flybase FB2021_04). Below the genomic organization of four different transcripts (erm-RA to erm-RD) are shown (white untranslated region; blue, translated region). These transcripts are translated into various protein forms using an ATG in exon 2 (erm-PA, erm-PC, erm-PD) or exon 3 (erm-PB). At the bottom is the genomic organization of the erm^KOGal4^ strain. Here, the region upstream of the ATG in exon 2 up to sequences shortly downstream of the ATG in exon 3 is deleted (black arrowheads) and replaced by Gal4 (yellow) flanked by an attB/P site (red) and a loxP site (green) as shown by [38]

**Fig. 2:**
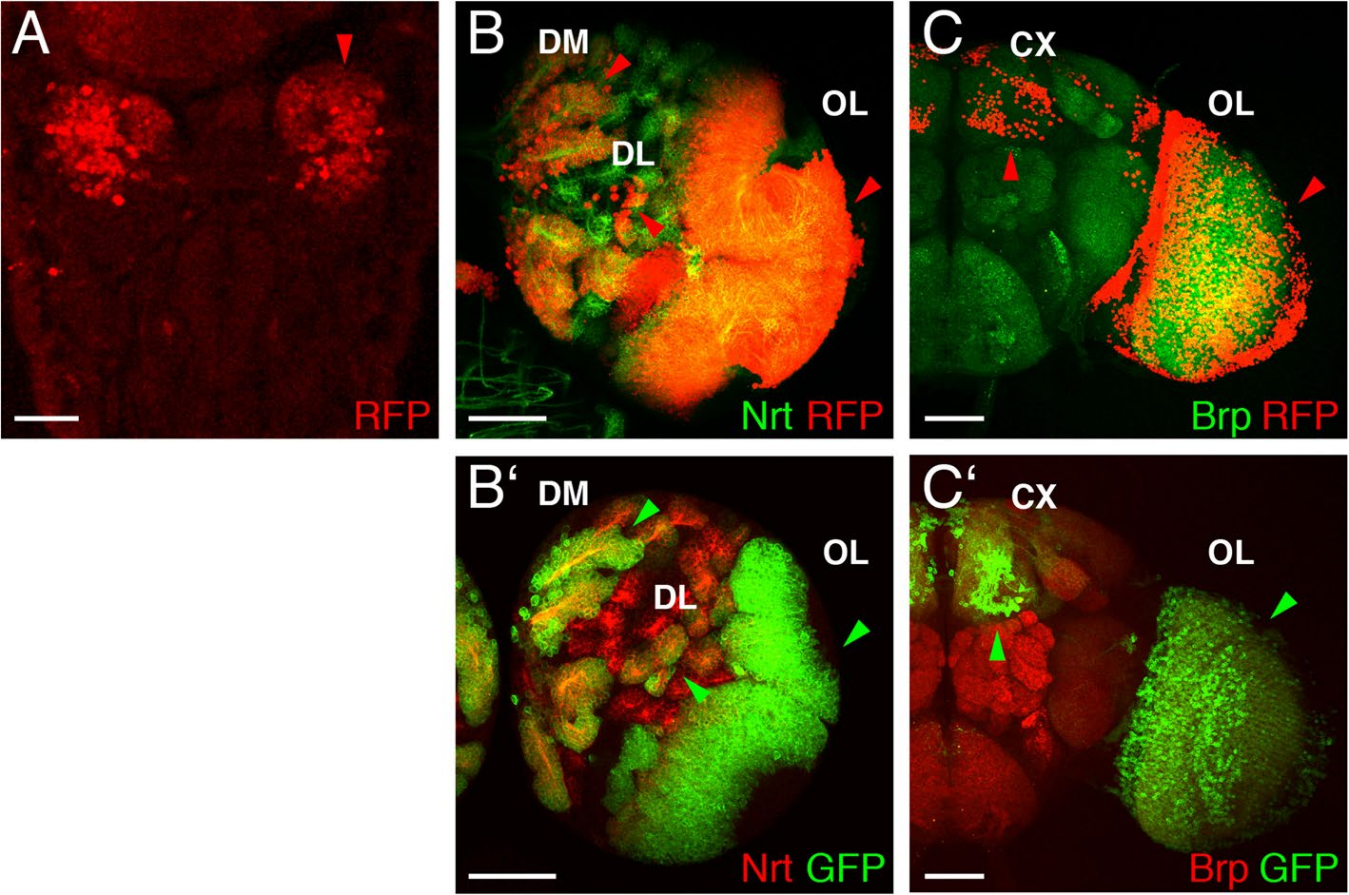
Expression of the erm^KOGal4^ strain. Laser confocal images showing the expression of the erm^KOGal4^ strain in different developmental stages visualized using an UAS-H2B-mRFP1 strain. **A** In a stage 16 embryo (the anterior end of the embryo is pointing down), erm^KOGal4^ dependent marker RFP expression is shown in red. Strong expression of the nuclear marker RFP was observed in the embryonic brain (red arrowhead). **B** In the right hemisphere of a third instar larval brain, Nrt staining (green) was used to highlight secondary neurons, erm^KOGal4^ marker expression in the type II lineages and the optic lobe is shown in red (red arrowheads). **C** The right part of an adult brain is visualized using Brp staining to mark the neuropile in green, and erm^KOGal4^ marker expression is again shown in red, here in the optic lobe and the central complex (red arrowheads). CX, central complex; DL, dorsolateral lineages; DM, dorsomedial lineages; OL, optic lobe. B’ larval brain with Nrt (red) and mCD8::GFP (green). C′ adult brain with Brp (red) and mCD8::GFP (green). (Scale bars: A, 25 μm; B-C′, 50 μm)

Despite the existence of various *erm* enhancer constructs recapitulating various aspects of *erm* expression, it would be good to have an *erm* enhancer trap strain that recapitulates the complete *erm* expression pattern. Additionally, this strain should also inactivate the *erm* gene function in homozygous flies. Under ideal circumstances, *erm* enhancers should activate Gal4 reintegrated in the locus to use such a strain as an enhancer trap strain and for overexpression, downregulation and rescue experiments using the UAS/Gal4 system [37]. To follow up on this idea, we used the gene targeting vector pTV^cherry^ [38], which is suitable for this experimental design. With this vector, it is possible to generate a deletion in the *erm* locus to inactivate the gene by gene targeting and at the same time integrate an attP site in the locus. With the use of a reintegration vector, it is then possible to integrate Gal4 in the locus. The *erm* gene has three exons and four different transcript forms generating four different protein forms varying in the N- and C-termini (Flybase FB2021_04). For the three protein forms the ATG resides in exon 2 (RA, RC, RD), and for the fourth form resides in exon 3 (RB). To inactivate the gene completely, we decided to delete a region of 1507 bp starting 9 bp upstream of the ATG in exon 2, including the intron between exons 2 and 3, and to remove the ATG in exon 3 (Fig. 1, black arrowheads). We amplified and cloned two 2.7 kb homologous regions flanking the area to be deleted in the pTV^cherry^ vector, made transgenic fly lines and mapped their chromosomal position. Among 24,625 flies that were the offspring of our gene targeting crosses we identified 13 red eyed flies resulting in a gene targeting frequency of 1/1894. Some of these flies were balanced and analysed by PCR to verify that the homologous recombination was correct. In one of the resulting *erm* targeting strains, erm^KO^, deleted *erm* sequences were replaced through the homologous recombination by a cassette including the *white* marker, loxP sites and an attP sequence [38]. With the use of the loxP sites, we removed the *white* gene and reintegrated Gal4 at the attP position with the help of the reintegration vector RIV^Gal4^ [38]. After selection of the correct transgenic flies, the *white* marker was removed via the flanking loxP sites so that in the final fly strain, Gal4 and some adjacent sequences are now replacing the deleted sequences of *erm* (Fig. 1). To analyse this strain which we named erm^KOGal4^, we visualized the Gal4 expression through crosses to the nuclear marker strain UAS-H2B-mRFP1. In the embryo, the expression of erm^KOGal4^ was as expected in the embryonic brain, resembling the mRNA expression pattern (Fig. 2A, red arrowhead; [4]). In the third instar larval brain, very strong expression was seen in the optic lobe and in the type II DM and DL lineages using either the nuclear RFP marker (Fig. 2B, red arrowheads) or the membrane-bound GFP marker (Fig. 2B’, green arrowheads). The expression in the adult brain was compared to Bruchpilot (Brp) which labels synapses and can be used to mark the neuropile. Expression was strong in the optic lobe but also visible in the central complex (Fig. 2C, red arrowheads, 2C’ green arrowheads). In summary the expression of erm^KOGal4^ recapitulates the expression of *erm* in all tissues where the gene is expressed. As expected, homozygous erm^KOGal4^ animals die as third instar larvae showing an overgrowth phenotype in the brain typical of already known *erm* alleles such as *erm*^1^ and *erm*^2^ [3].

### Generation of an Erm antibody

To analyse Erm expression in our future experiments we decided to generate a new Erm antibody. We expressed the 130 carboxy-terminal amino acids of the protein forms erm-RA and erm-RB as a GST fusion protein in *E. coli*. We immunized a rabbit with our purified GST fusion protein and tested the resulting antibody. The antibody detected the Erm protein in the embryo in the already known expression domains starting from stage 5 with the typical earmuff pattern up to later stages with expression in the embryonic brain (Fig. S1). Unfortunately, the antibody showed no staining in larval stages or later (data not shown). Here an additional purification of the antibody or the use of staining techniques improving the sensitivity might help in the future.

### Analysis of *erm* enhancer constructs using the nuclear marker RFP

**Fig. 3:**
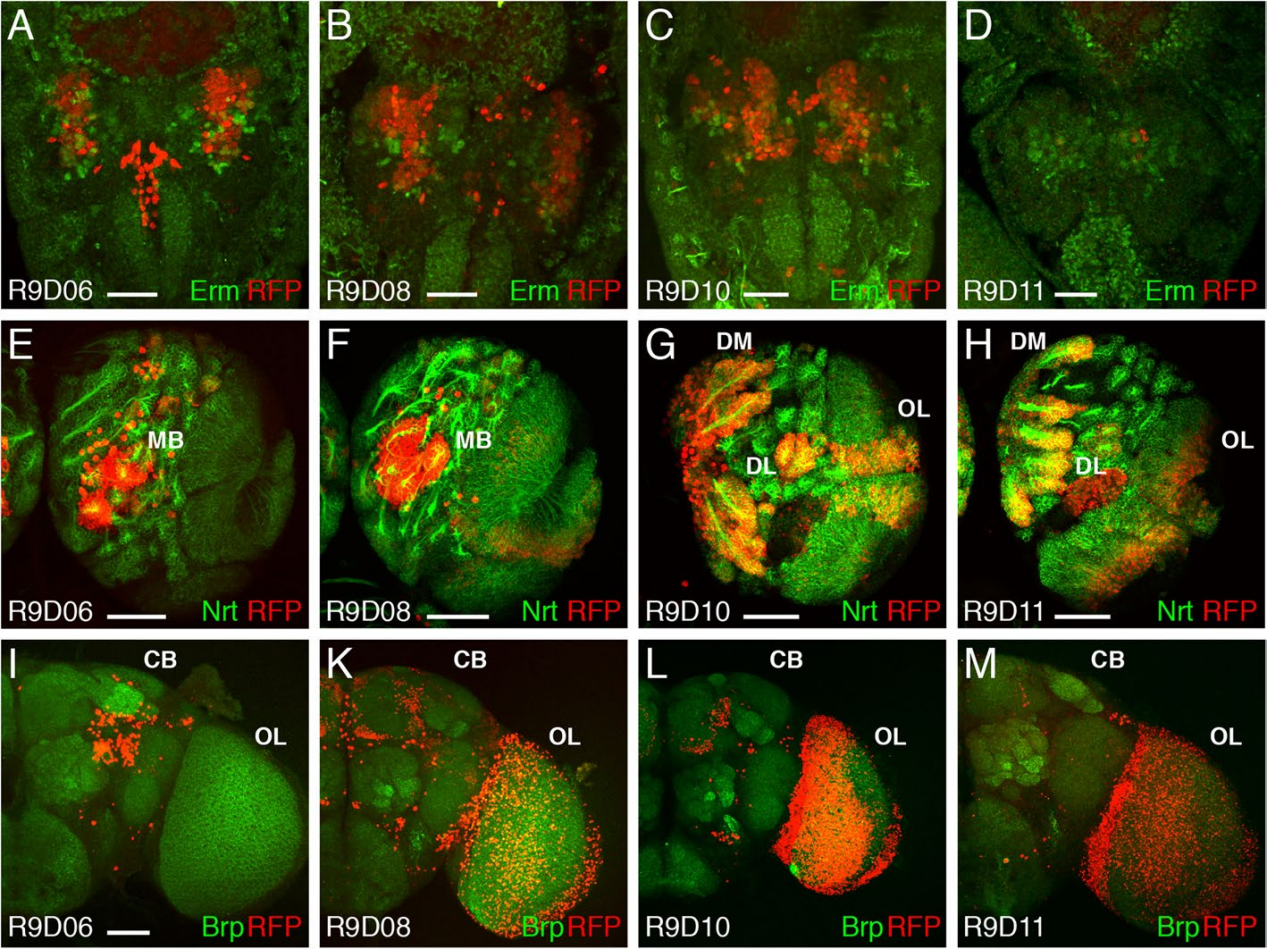
Expression of *erm* enhancer-Gal4 strains in embryos, larvae and adults. Laser confocal images of *Drosophila* embryonic (**A-D**), larval (**E-H**) and adult brains (**I-M**). For the embryos, only the anterior part with the brain is shown, and the anterior end is pointing down. Only the right hemispheres of the larval brains and the right sides of adult brains are shown. **A-D** Embryos labelled with an anti-Erm antibody (green) and Gal4 dependent nuclear RFP marker expression (red). **E-H** Larval brain hemispheres labelled with an anti-Nrt antibody to highlight secondary neurons (green) and Gal4 dependent nuclear RFP marker expression (red). (I-M) Adult brain hemispheres labelled with an anti-Brp antibody (green) and Gal4 dependent nuclear RFP marker expression (red). CB, central brain; DL, dorsolateral lineages; DM, dorsomedial lineages; MB, mushroom body; OL, optic lobe. (Scale bars: A-D, 25 μm; E-M, 50 μm)

With the help of enhancer-Gal4 constructs, a 20 kb region of the *erm* gene including 11 kb upstream and 5 kb downstream, was already analysed by in situ hybridization in the embryo and using mCD8::GFP in later stages to visualize the enhancer activity via GFP expression [4]. Several regions were identified driving expression in well-defined regions in the embryonic, larval, and adult brain. Prominent expression in the larval brain was observed with constructs R9D03, R9D04, R9D06, R9D08, R9D10 and R9D11 [4]. Constructs R9D03 and R9D04 with DNA from the 3′ region of the *erm* gene showed some expression in the adult optic lobe. The other four constructs cover the largest intron region of *erm*; here, R9D06 shows expression in the mushroom bodies of the larval brain, and R9D08 is also expressed in the mushroom bodies, but additionally in the interhemispheric bridge. The most prominent expression pattern was seen with R9D10 in the DM and DL lineages as well as in the optic lobe of larvae and in the fan-shaped body of the central complex in the adult brain. R9D11 shows the adult fan-shaped body expression but slightly weaker expression in the DM and DL lineages of the larval brain and very weak expression in the optic lobe. Recently it was shown that R9D11 also drives expression in embryonic optic neuroblasts (EONs), a newly discovered stem cell population generated from the optic lobe neuroepithelium [39]. To reanalyse the expression of the intronic constructs R9D06, R9D08, R9D10 and R9D11, we recombined the respective Gal4 constructs with an UAS-H2B-mRFP1 strain to generate strains permanently expressing the nuclear RFP marker. All of these strains were analysed in the embryonic, larval and adult brains. In R9D06, R9D08 and R9D10 embryonic brains, expression was visible in defined regions of the brain (Fig. 3A-C), whereas R9D11 showed only expression of RFP in a few cells, most likely 8–9 embryonic optic neuroblasts [39] (Fig. 3D). In R9D06 and R9D08 larval brains, reporter expression was observed in the mushroom body (Fig. 3E, F), and in R9D08 larvae, weak optic lobe expression was detected (Fig. 3F). R9D10 and R9D11 showed strong RFP expression in the type II DM and DL lineages and in the optic lobe (Fig. 3G, H). In the adult brain, R9D06 was expressed in the mushroom body (Fig. 3I), and R9D08 and R9D10 were expressed in the central complex (Fig. 3K, L). Reporter expression in the adult optic lobe was present in R9D08, R9D10 and R9D11 (Fig. 3K-M), and was strongest in R9D10 (Fig. 3L).

### Generation of *erm* enhancer deletions by gene targeting

**Fig. 4:**
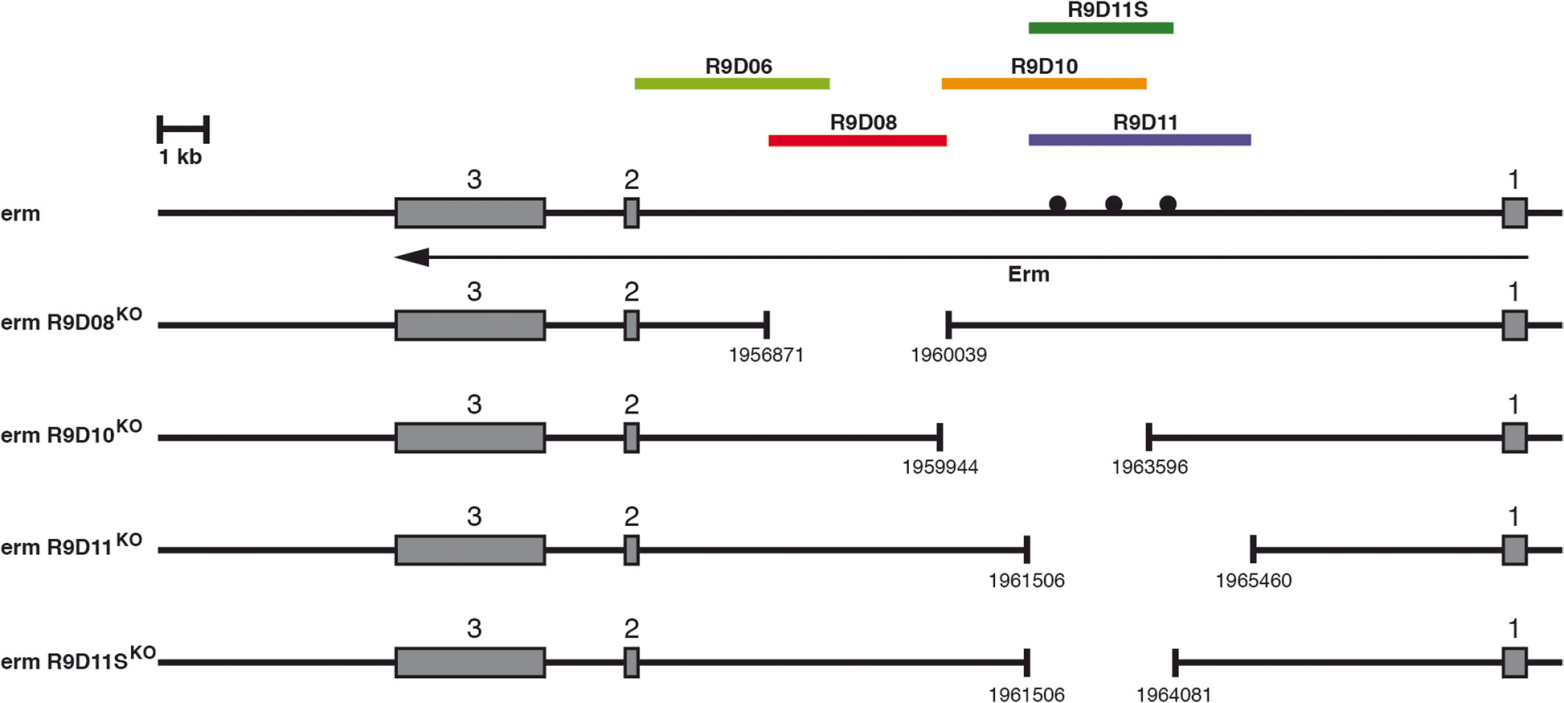
Deletions of *erm* enhancers by gene targeting. The genomic organization of the *erm* gene with exons indicated as grey boxes is shown together with the location of the analysed enhancers. Binding positions of bHLH-O proteins such as Deadpan and Enhancer of split proteins are shown as black circles. Below the genomic organization of the individual enhancer deletion strains is indicated. Deletion breakpoint positions are indicated according to the sequences from Flybase (FB2021_04)

We planned to generate constructs deleting individual enhancers by gene targeting via homologous recombination. We decided to delete the genomic regions present in the constructs R9D08, R9D10 and R9D11. We did not delete R9D06 since the expression in the mushroom body is also present in the overlapping construct R9D08. For R9D11, it was shown that in the more proximal region, bHLH-O proteins such as Deadpan and Enhancer of split proteins bind and thereby regulate *earmuff* [26]. We therefore decided to remove the more distal part of R9D11, reducing the deleted region from 3.9 kb to 2.5 kb in the smaller targeting construct R9D11S (Fig. 4). Similar to the *erm* gene targeting construct, we again PCR amplified 2.7 kb homology arms, cloned them in the pTV^cherry^ vector, made transgenic flies and generated targeting flies through the appropriate fly crosses. In the case of R9D08GT, we screened 25,029 flies and identified 19 red-eyed flies (1/1317); for R9D10GT, 30,219 flies and identified 27 red-eyed flies (1/1119); for R9D11GT, 56,268 flies and identified 27 red-eyed flies (1/2084); and for R9D11SGT, 21 red-eyed flies among 38,396 flies were recovered (1/1828). In all cases the *white* gene was removed and the final strains R9D08^KO^, R9D10^KO^, R9D11^KO^ and R9D11S^KO^ were molecularly analysed by PCR and sequencing of the deletion breakpoints. All four strains showed the expected deletions and were balanced for further analyses.

### Functional analysis of *erm* enhancer deletion strains

**Fig. 5:**
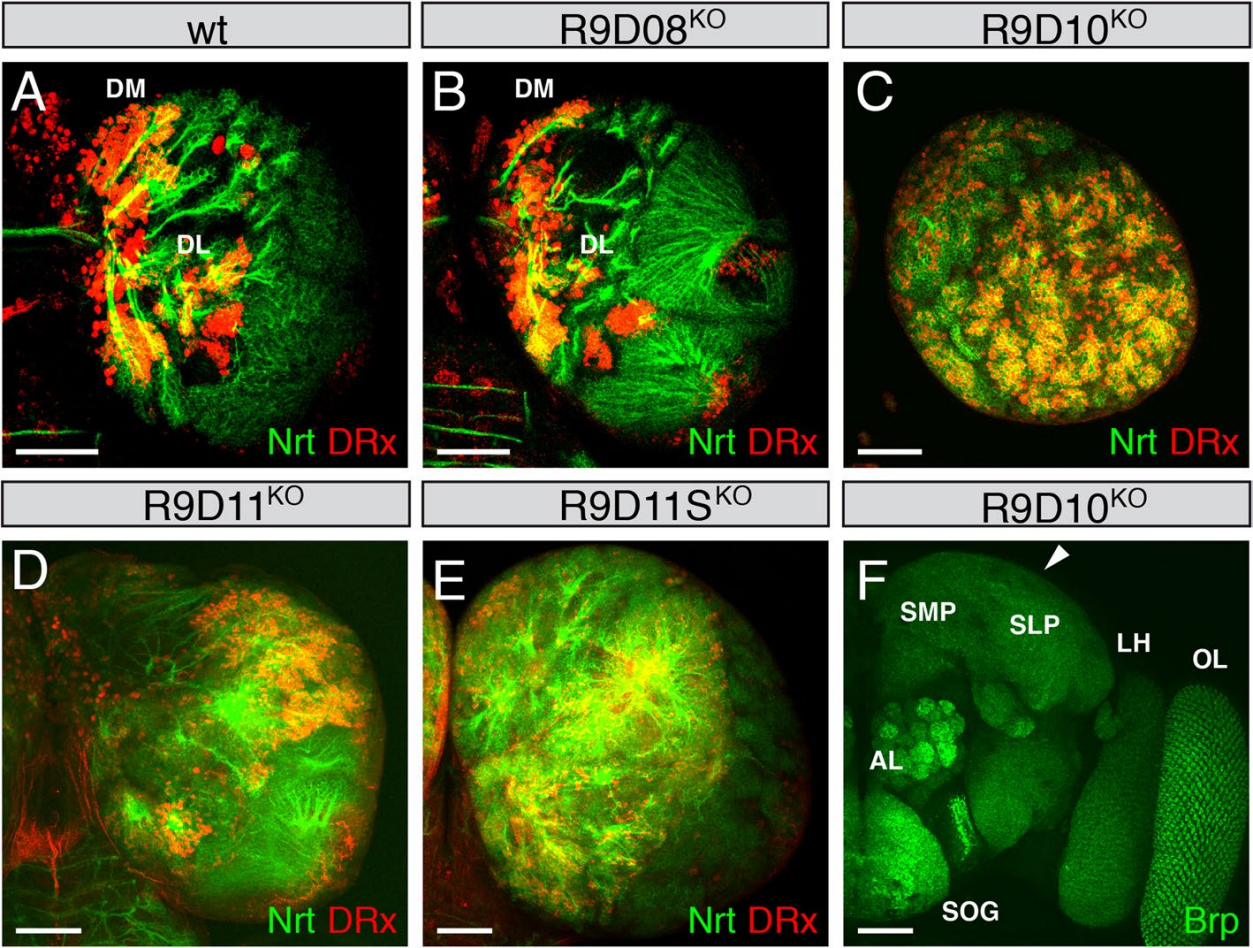
Phenotypic analysis of *erm* enhancer deletion strains. Laser confocal images of larval and adult brain hemispheres from *erm* enhancer deletion strains labelled with anti-Nrt to visualize secondary neurons and anti-DRx as a marker showing expression in the type II lineages, the mushroom bodies and in the optic lobe in larval brains and anti-Brp to visualize the adult brain. The right larval brain hemisphere and the right part of an adult brain is always shown. For all *erm* enhancer deletion strains homozygous animals were analysed. **A** Wild-type larval brain hemisphere showing the DRx expression in DM and DL lineages as well as in the optic lobe. **B** Strain R9D08^KO^ shows a more or less identical staining pattern relative to the wild-type strain. **C** In strain R9D10^KO^, the brain hemisphere was enlarged and showed more lineages, and the DRx expression was increased throughout the hemisphere. **D, E** In strains R9D11^KO^ and R9D11S^KO^ the brain hemispheres were also enlarged and looked disorganized but showed less DRx staining than to R9D10^KO^ (**C**). **F** Right part of an adult brain of strain R9D10^KO^ showing an enlargement of the superior lateral protocerebrum (white arrowhead). Abbreviations: AL, antennal lobe; DL, dorsolateral lineages; DM, dorsomedial lineages; LH, Lateral Horn; OL, Optic Lobe; SOG, Suboesophageal Ganglion; SLP, Superior Lateral Protocerebrum; SMP, Superior Medial Protocerebrum. (Scale bars: 50 μm)

First, the lethality was analysed for all strains. Strain R9D08^KO^ is not lethal, whereas in strain R9D10^KO^ most animals die as third instar larvae, but a few escapees develop to the adult stage. In strains R9D11^KO^ and R9D11S^KO^ all animals died as third instar larvae. We next analysed larval brains of all four strains using the neuronal marker Nrt [40] to visualize all secondary neurons in the larval brain. In addition, we analysed the expression of the homeodomain transcription factor DRx which is expressed in all type II lineages of the larval brain [41, 42] as well as in the optic lobe [43] (Fig. 5A). In larval brains from strain R9D08^KO^ no obvious alterations of the DRx expression pattern were visible (Fig. 5B) compared to the wild-type (Fig. 5A). In contrast in strain R9D10^KO^ the brain size was enlarged, more lineages were established, and the expression of DRx was dramatically increased (Fig. 5C). Additionally, in strains R9D11^KO^ and R9D11S^KO^ the brains were enlarged and lineages were disorganized (Fig. 5D, E), but the increase of DRx expressing cells was less pronounced than that in R9D10^KO^ (Fig. 5C). The enlargement of the brain sizes of R9D10^KO^, R9D11^KO^ and R9D11S^KO^ animals was between 20 and 30% (compare scale bars in Fig. 5C-E to Fig. 5A). This demonstrates that the common region deleted in these three strains is responsible for this phenotype. In the adult brain of strain R9D10^KO^, an enlargement was visible in the region of the superior lateral protocerebrum (Fig. 5F, white arrowhead). Phenotypes in other regions specific for the individual deletions might be present but could not be shown by this analysis. This could be analysed in the future using these *erm* enhancer deletion strains with more specific markers.

## Discussion

In this paper we analysed enhancers of the *erm* gene and generated deletions of important *erm* enhancers by gene targeting, providing the basis for a much deeper functional analysis of the *erm* enhancers and various processes in which Erm is involved. The best analysed function of Erm is its function during INP maturation [3, 13]. Here, it works together with the SWI/SNF chromatin-remodelling complex Brahma [21, 44–46]. The developmental potential of neural progenitor cells in type II lineages underlies a timely restraint depending on the rapid activation of Earmuff. This is achieved by a poising and activation mechanism [21]. Here Rpd3 maintains the poised *erm* immature enhancer inactivity in neuroblasts [21]. Through a rapid downregulation of self-renewing factors and the acetylation of histone proteins Erm expression in immature INPs will be activated. Another factor interacting with Erm is Notch, which maintains type II neuroblasts by suppression of Erm via the Ets family transcription factor Pointed P1 (Pnt P1) [47]. The balance between self-renewal and differentiation is also regulated by the bHLH-O protein Deadpan and some Enhancer of Split proteins [48, 49], which bind to C-sites and N-boxes in the enhancer region R9D11 of *erm* to suppress the Erm expression [26]. Additionally, the *Drosophila* integrator complex [50, 51] prevents INP dedifferentiation by regulating of Erm [52]. Recently, it was shown, that Six4 is yet another factor preventing the generation of supernumerary type II neuroblasts through the formation of a trimeric complex with Earmuff and Pointed P1 [53].

Earmuff also has a function in the fly visual system where it is expressed specifically in L3 neurons of the lamina as shown by cell sorting experiments followed by RNA-seq analysis [54]. The L3 neurons innervate M3, one of the six outer layers of the medulla [55]. Erm controls this innervation via the expression of DRP proteins [56] which mediate interactions with medulla target cells [57]. In addition, it controls the layer specificity of R8 through the activation of the secreted protein Netrin [58] in L3 neurons [57]. In L3 neurons not only cell surface molecules but also transcription factors belong to the two largest groups that are differentially expressed in *erm* null L3 neurons. One of these transcription factors is Sloppy-paired1 (Slp1) [59], known for its embryonic function as a regulator involved in segmentation. In this context, in L3 neurons, Erm functions as a transcriptional repressor, since in *erm* null L3 neurons, Slp1 is upregulated, leading to improper target recognition of L3 growth cones [60].

The analysis of *erm* enhancers started with a systematic analysis of enhancers of genes with expression in the brain [4]. In this study, *erm* was one of four genes analysed in detail. In total nine enhancer fragments were analysed, and only fragment R9D05 in the intron of the *erm* gene showed no expression [4]. This is exactly the region between exons 2 and 3 that is deleted in our erm^KOGal4^ targeting strain. Therefore, we would not expect the loss of an *erm* enhancer in our strain. Pfeiffer et al. found that 80% of all 44 fragments tested showed expression in the brain. This is even more than that reported in a more recent analysis where 46% of 7705 tested fragments were active in the embryo [61]. These analyses suggest that 50,000 to 100,000 enhancers exist in the *Drosophila* genome [4, 61]. Among these enhancers are redundant enhancers, called shadow enhancers which provide robustness to regulatory networks [62, 63]. Among the *erm* enhancers, there are most likely no shadow enhancers, since all enhancer fragments drive expression in well-defined regions, and if two enhancer fragments show a similar pattern they are at least partially overlapping. Our erm^KOGal4^ targeting strain seems to reflect the normal expression pattern of *erm* during development. Recently, another *erm*-Gal4 strain (18B02 dFezf-GAL4) was generated using the BAC CH321-18B02 (BACPAC Resources Center), including the *erm* locus. Through the recombineering technique [64] the coding region of *erm* was replaced by Gal4 and the construct was inserted in the VK33 site on the third chromosome [60]. The construct mimics Erm expression in L3 neurons [61]. We would expect that this strain and our strain will both recapitulate the *erm* expression similarly, but with our erm^KOGal4^ strain, it will also be possible to analyse the reporter gene expression on an *erm* mutant background at least up to the larval stage.

Using gene targeting technology and the vector pTV^cherry^ [38], we generated five donor constructs to generate *erm* strains with deletions of four different enhancer regions and one strain with a deletion of the N-terminal part of the *erm* coding region to reintegrate Gal4 at that position. The targeting efficiency in these experiments depends on various parameters. Important is the length of the homology arms used for homologous recombination and the size of the region to be deleted. Here, longer homology arms and shorter deletions enhance the efficiency. The initial use of the pTV^cherry^ vector by Baena-Lopez et al. resulted in targeting efficiencies between 1/1000 and 1/3000 in five cases and 1/8000 in one case using homology arms between 3 and 5 kb length. We performed our first targeting experiments with pTV^cherry^ with 4.0 kb homology arms and deleted the N-terminal part of the *hbn* gene (Hildebrandt et al., in preparation) and of the *DRx* gene [42] with an efficiency between 1/600 and 1/700 which was even better than that previously reported [38]. This might be a consequence of the rather small deletions made which are between 0.17 kb and 0.39 kb. To generate the 1.5 kb deletion of the N-terminal part of the *erm* coding region including the intron we reduced the homology arms to 2.7 kb, resulting in a drop in the efficiency to 1/1894. For the enhancer deletions that were between 2.5 kb and 3.9 kb, a similar targeting efficiency was observed (1/1317–1/2084). These results indicate that homology arms of 2.7 kb used to generate deletions up to 3.9 kb result in good efficiencies and might be good choices for future targeting experiments using the pTV^cherry^ vector.

Our analysis of the *erm* enhancer gene targeting strains showed phenotypes that are all typical for a loss of *erm* function but could now be assigned to specific enhancers. A loss of *erm* function in larvae shows enlarged brain lobes with a tenfold increase in neuroblasts compared to wild-type larvae [3]. The R9D08^KO^ strain shows no DRx expression in the region of the mushroom bodies arguing for a loss of this structure. In strain R9D10^KO^ the presence of additional neuroblasts and lineages is most prominent in the optic lobe, and in R9D11^KO^ and R9D11S^KO^, the type II lineages are more affected. In future experiments it will be possible to analyse the enhancer deletions also in earlier stages using more specific markers to see when the phenotypes start to be visible and at which timepoint of development they are completely established.

## Conclusion

Earmuff expression is regulated by several well defined enhancer regions. We generated a new *erm*-Gal4 strain with the possibility of analysing *erm* expression on an *erm* mutant background. Through the generation of *erm* enhancer deletions by gene targeting, we identified the first phenotypic alterations that could be assigned to specific enhancer regions. Our experiments provide the basis for a much deeper functional analysis of *earmuff* enhancers and various Erm-regulated processes in the future.

## Methods

### Fly strains

The following fly strains were used: yw67c3; UAS-H2B-mRFP1, UAS-mCDC8-GFP [65]; ubiquitin-Gal4[3xP3-GFP] [38]. The following stocks were obtained from the Bloomington Drosophila Stock Center (BDSC):

y [1] w[67c23]; sna[Sco]/CyO, P{w[+mC] = Crew}DH1 (BL 1092);

y [1] w[*]/Dp(2;Y)G, P{w[+mC] = hs-hid}Y; P{ry[+t7.2] = 70FLP}23 P{v[+t1.8] = 70I-SceI}4A/TM3, P{w[+mC] = hs-hid}14, Sb [1] (BL 25679);

y [1] w[*] P{y[+t7.7] = nos-phiC31\int.NLS}X; sna[Sco]/CyO (BL 34770);

w[1118]; P{y[+t7.7] w[+mC] = GMR9D10-GAL4}attP2 (BL 40730);

w[1118]; P{y[+t7.7] w[+mC] = GMR9D11-GAL4}attP2 (BL 40731);

w[1118]; P{y[+t7.7] w[+mC] = GMR9D08-GAL4}attP2 (BL 47424);

w[1118]; P{y[+t7.7] w[+mC] = GMR9D06-GAL4}attP2 (BL 65401).

### Antibody production

To generate anti-Erm antibodies against the Erm C-terminus a 0.4 kb fragment was amplified using BAC CH322-100 J13 DNA [66] and the primers ermAK3 (5′-TATAGAATTCGATCGGCGGCGATC-3′) to add an *Eco*RI site (underlined) and ermAK2 (5′-TATAGTCGACGCTGTCAAAACACCTTGGCTATGA-3′) to add a *Sal*I site (underlined). The fragment was subcloned into the vector pCRII-TOPO (Invitrogen, Carlsbad, California, USA), cut out with *Eco*RI and *Sal*I as a 410 bp fragment and cloned in frame into the pGEX-4 T1 expression vector (Amersham, Buckinghamshire, United Kingdom). The fusion protein of glutathione-S-transferase and Erm was purified as described [67]. Immunization of a rabbit was performed by the Medical Biochemistry Department of the Saarland University (Homburg, Germany).

### Immunostaining

Embryos were collected, dechorionated with 50% bleach for 2 min, washed with 0.1% NaCl /0.1% Triton X-100 and fixed for 12 min in 3.7% formaldehyde in PEM (100 mM PIPES, 1 mM EGTA, 1 mM MgCl_2_) and heptane. After removal of both phases, embryos were devitelinized in equal volumes of heptane and methanol by 2 min of vigorous shaking and washed three times with methanol. The 3rd instar larvae and adult brains were dissected in 1x phosphate buffered saline (PBS), fixed for 60 min in 2% paraformaldehyde in PBL, washed three times with 1x PBS containing 0.2% Triton X-100 (PBX) and then incubated for 3 × 5 min in methanol.

For alkaline phosphatase antibody stainings fixed embryos were washed 3 × 20 min in PBT (1xPBS, 0.2% Tween20) and blocked for 30 min in TNB (0.1 M Tris pH 7.5, 0.15 M NaCl, 0.5% (w/v) blocking reagent) Incubation with the primary antibodies were performed overnight at 4 °C. Embryos were washed 3 × 5 min and 3 × 20 min in PBT and blocked for 30 min in TNB. After an overnight incubation with an AP-conjugated secondary antibody at 4 °C embryos were washed 3 × 5 min and 6 × 20 min in PBT, 2 × 10 min in AP buffer (0.1 M Tris pH 9.5, 0.1 M NaCl, 50 mM MgCl_2_, 0.1% Tween20). To 1 ml AP buffer 9 μl NBT (50 mg/ml) and 3.5 μl BCIP (50 mg/ml) were added for the staining reaction. Stained embryos were incubated 3 × 2 min in PBT, dehydrated in an ethanol series and mounted in Canada balsam. For fluorescence stainings fixed embryos or larvae were washed 3 × 5 min and 6 × 30 min in PBX and blocked for 30 min in 5% normal horse serum and 10% PBX in PBS. Incubations with primary antibodies were performed overnight at 4 °C. Samples were washed 3 × 5 min and 6 × 30 min in PBX and blocked for 30 min in 5% normal horse serum and 10% PBX in PBS. After an overnight incubation with secondary antibodies at 4 °C embryos or larvae were washed 3 × 5 min and 6 × 30 min in PBX and mounted in Vectashield (Vector Laboratories). Adult brains were treated the same as larval brains but were incubated with the appropriate antibody two nights each. Images were obtained using an Olympus BX61 microscope (Olympus, Hamburg, Germany) for bright field and DIC microscopy or a Leica TCS SP5 microscope (Leica, Wetzlar, Germany) for laser confocal microscopy. Images were processed using FIJI and ImageJ (NIH. Md., USA), Adobe Photoshop and Adobe Illustrator (Adobe Systems, San Jose, CA, USA).

The primary antibodies used were rabbit anti-DRx antibody (1:1000) [68], rabbit anti-Erm antibody (1:200, this paper), mouse anti-Nrt (BP106) antibody (1:25) (DSHB) and mouse anti-Brp (nc82) antibody (1:25) (DSHB). The secondary antibodies were goat anti-mouse and anti-rabbit conjugated with Alexa 488 or 568 (1:1000, Molecular Probes, Eugene, Oregon, USA) and alkaline phosphatase conjugated goat anti-rabbit (1:1000, Jackson Immuno Research).

### Generation of an *erm* gene targeting construct

An *erm* donor gene targeting construct was made in the vector pTV^cherry^ according to [38]. The two 2.7 kb homology arms were amplified using Pfu DNA Polymerase (New England Biolabs) and BAC CH322-100 J13 DNA [66]. The primers ermGT1 (5′-TATACCGCGGAATCCCGAAGTGACCTTTAACCC-3′) and ermGT2 (5′-TATAGGTACCTGCCTATGTGGATATCCAG-3′) were used for homology arm 1 and ermGT3 (5′-TATAACTAGTCGCCTTCGAAGAGCCCCGTG-3′) and ermGT4 (5′-TATAGGCGCGCCTTAGGATCCCTCCACTCGACTC-3′) were used for homology arm 2. All primers came with unique restriction enzyme recognition sites added to their ends (underlined), which enabled later cloning in the final vector. After adding 3′ adenine overhangs to the PCR products they were subcloned into the vector pCR 2.1 (Thermo Fisher Scientific, Waltham, Massachusetts, USA) and checked by sequencing. In the correct clones, the homology arms were cut out with the relevant restriction enzymes and finally cloned in the vector pTV^cherry^ [38]. P-element-mediated transformation into *yw*^*67c23*^ flies was performed by BestGene (Chino Hills, California, USA). Transformants were balanced and transformants with integration on the third chromosome were used for the generation of final targeting strain. Transformants were crossed to *hs-Flp, hs-SceI* flies (BL 26579) and the resulting larvae were heat-shocked 48 h and 72 h after egg laying for 1 h at 37 °C. Two hundred adult female flies with mottled red eyes were crossed with *ubiquitin-Gal4[3xP3-GFP]* males, and the progeny were screened for the presence of red-eyed flies. The transgene *ubiquitin-Gal4[3xP3-GFP]* was removed by selection against GFP expression and the resulting targeting flies were balanced over CyO and molecularly analysed for the correct integration event. To verify this, we performed PCRs with primers within the cassette brought in by the homologous recombination events and primers located outside of the homology arms (ermGT1A (5′-GATGGGTTAAGGTAGTACCAAGC-3′) and mCherryrev2 (5′-CCTCGTCGTCGTTCAGGTTG-3′) for the upstream region and ermGT4A (5′-CTTGGGCCCGAGTAATGCAGC-3′) and pTVGal4–1 (5′-CGTTTTTATTGTCAGGGAGTGAGTTTGC-3′) for the downstream region. From one of these strains, called erm^KO^, removal of the white gene was performed by crossing of the *erm-*targeting flies to a strain expressing Cre Recombinase (BL 1092) and selecting and balancing of white eyed flies in the offspring of the crosses. For the reintegration of Gal4 in the *erm* locus, the vector RIV^Gal4^ was used [38]. *Erm*-targeting flies were crossed with PhiC31-expressing flies (BL 34770) and embryos of that cross injected with RIV^Gal4^ DNA. Red-eyed transformant flies were selected and the *white* marker was removed again using the loxP sites to generate the strain erm^KOGal4^.

### Generation of *erm* enhancer deletions by gene targeting

Erm donor constructs for the deletion of enhancer regions were made in the same way as was described for the *erm* gene targeting construct using BAC CH322-66P22 and CH322-100 J13 DNA [66]. In all cases homology arms of approximately 2.7 kb were PCR amplified using GT1 and GT2 primers for homology arm 1 and GT3 and GT4 primers for homology arm 2. The following primers were used: For the R9D08GT construct, the primers R9D08GT1 (5′-GCGGCCGCTTAGTTGGGTTCGAAGTAAACAGAG-3′), R9D08GT2 (5′-GGTACCTTGGGGTTGGGGGATGACTAC-3′), R9D08GT3 (5′-AGATCTCTCTGAACTCTGCGCCATTTGC-3′) and R9D08GT4 (5′-GGCGCGCCTGCTTTTCTTACGAACGTCACG-3′); for the construct R9D10GT, the primers R9D10GT1 (5′-GCGGCCGCGACAGTCGGCAAATTACCAGTCG-3′), R9D10GT2 (5′-GGTACCCTGACATTTGATTTCGCTTTCGGC-3′), R9D10GT3 (5′-ACTAGTGGGGTGTGTAATCCTGTCGAGG-3′) and R9D10GT4 (5′-GGCGCGCCGACTGCTCTACTAAACCAATAAAACG-3′); for the construct R9D11GT, the primers R9D11GT1 (5′-GCGGCCGCAACTCGCCTGGGAATCG-3′), R9D11GT2 (5′-GGTACCTAATCCAAAGGTGGCGGGTTC-3′), R9D11GT3 (5′-ACTAGTTTGGTTAGCCGCAGAAATTGACC-3′), and R9D11GT4 (5′-GGCGCGCCACGTGCTTGTGCATTTCACTCTC-3′). For the construct R9D11S, the primers R9D11GT8 (5′-GCGGCCGCAACGTTCTGTATGTAGGAATATCCTAGAGAAG-3′) and R9D11GT9 (5′-GGTACCAGCAAGAAGTTCTGCCTCTTCTTG-3′) were used for homology arm 1, and homology arm 2 was the same as for the R9D11 construct. To prove that the deletions were as predicted we PCR amplified the deletion breakpoints and sequenced the PCR products.

## Supplementary Information

**Additional file 1: Figure S1.** Spatial distribution of Erm during *Drosophila* embryogenesis. Antibody stainings of wild-type embryos using an anti-Erm antibody. Stages were determined according to [69] and are indicated in the figure. All views are dorsal views, anterior is to the left. Expression was detected in the procephalic ectoderm (A-C, black arrowheads), from stage 11 on in the brain (D-F, black arrowheads) and in the hindgut (D, white arrowhead). (Scale bar: 50 μm).

## Data Availability

The datasets supporting the conclusions of this article are included within the article. Materials are available from the corresponding author upon reasonable request.

## Abbreviations

BDSC: Berkeley Drosophila Stock Center
BPRC: BACPAC Resources Center
DSHB: Developmental Studies Hybridoma Bank
