# Supplementary figures and images for: Enhancer analysis of the Drosophila zinc finger transcription factor Earmuff by gene targeting
Source: Hereditas. 2021 Nov 4;158:41. doi: 10.1186/s41065-021-00209-6 (PMC8567707; doi:10.1186/s41065-021-00209-6)

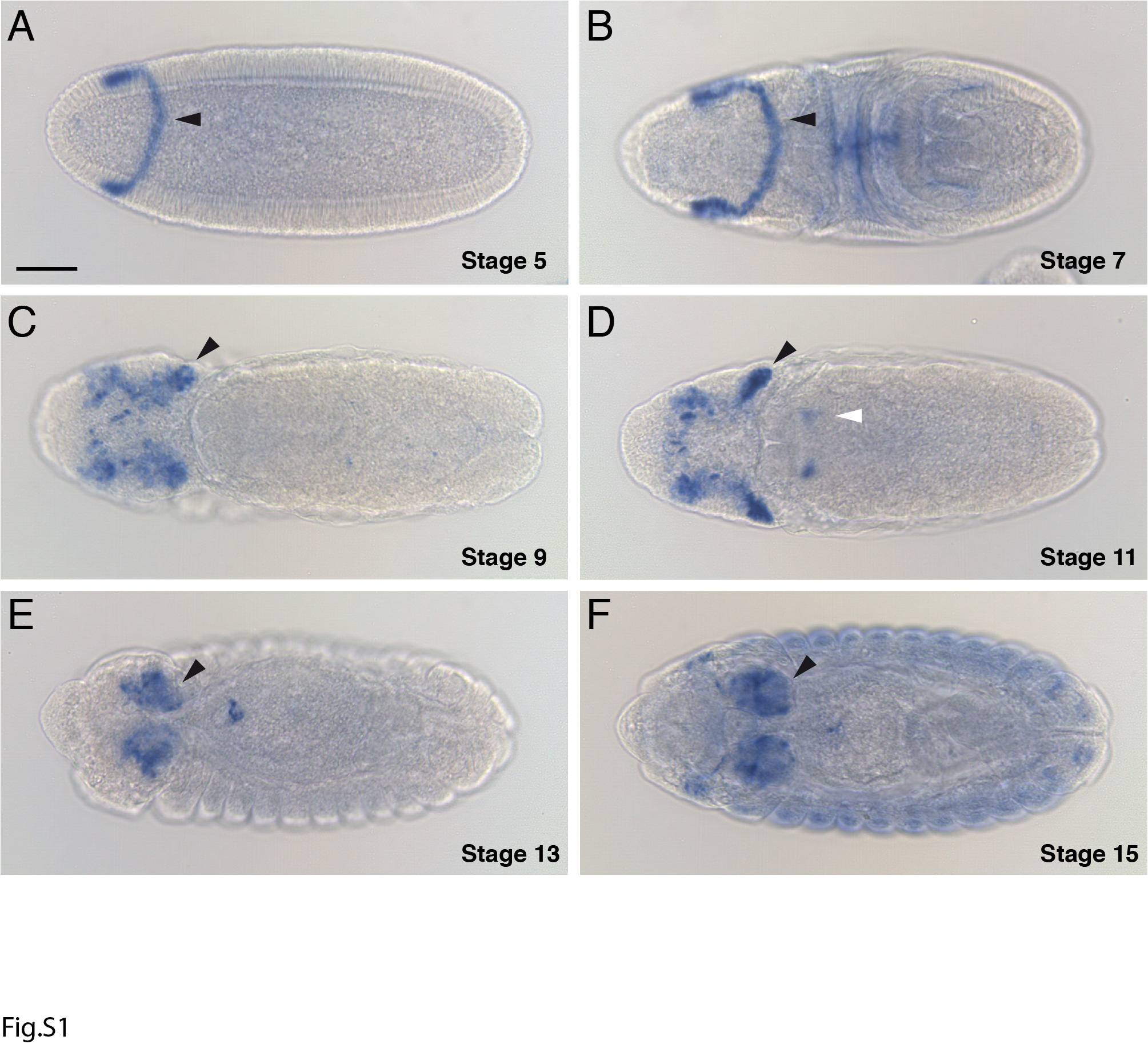

Supplement: Supplementary file 1 — Additional file 1: Figure S1. Spatial distribution of Erm during Drosophila embryogenesis. Antibody stainings of wild-type embryos using an anti-Erm antibody. Stages were determined according to [69] and are indicated in the figure. All views are dorsal views, anterior is to the left. Expression was detected in the procephalic ectoderm (A-C, black arrowheads), from stage 11 on in the brain (D-F, black arrowheads) and in the hindgut (D, white arrowhead). (Scale bar: 50 μm). [file 41065_2021_209_MOESM1_ESM.jpg]
